# Living strategy of cold-adapted fungi with the reference to several representative species

**DOI:** 10.1080/21501203.2017.1370429

**Published:** 2017-08-30

**Authors:** Manman Wang, Jianqing Tian, Meichun Xiang, Xingzhong Liu

**Affiliations:** ^a^ College of Life Science, Hebei University, Baoding, China; ^b^ State Key Laboratory of Mycology, Institute of Microbiology, Chinese Academy of Sciences, Beijing, China; ^c^ University of Chinese Academy of Sciences, Beijing, China

**Keywords:** Cold-adapted fungi, cold habitats, diversity, ecological function, living strategy

## Abstract

Our planet is dominant with cold environments that harbour enormously diverse cold-adapted fungi comprising representatives of all phyla. Investigation based on culture-dependent and independent methods has demonstrated that cold-adapted fungi are cosmopolitan and occur in diverse habitants and substrates. They live as saprobes, symbionts, plant and animal parasites and pathogens to perform crucial functions in different ecosystems. *Pseudogymnoascus destructans* caused bat white-nose syndrome and *Ophiocordyceps sinensis* as Chinese medicine are the representative species that have significantly ecological and economic significance. Adaptation to cold niches has made this group of fungi a fascinating resource for the discovery of novel enzymes and secondary metabolites for biotechnological and pharmaceutical uses. This review provides the current understanding of living strategy and ecological functions of cold-adapted fungi, with particular emphasis on how those fungi overcome the extreme low temperature and perform their ecological function.

## 1. Introduction

Cold ecosystems are dominant in our planet and the temperatures of almost 85% of earth is permanently or seasonally below 5°C ranging from deep sea to alpine and from Antarctica to Arctic regions (Hassan et al. 2016). Cold conditions, together with other limiting factors (e.g. low water and nutrient availability, high hydrostatic pressure and oxidative stress, high solar irradiation), have a strong influence on whether a certain organism can survive or even successfully thrive in a given habitat (Margesin and Miteva 2011).

The classical definitions based on thermal dependence of growth kinetic parameters (Morita 1975) are used to classify cold-adapted organisms. Accordingly, psychrophiles have been defined as species that can grow at or below 0°C, with optimum growth temperatures (OGTs) of ≤15°C and maximum growth temperatures (MGT) of ≤20°C, while psychrotolerants can grow close to 0°C, with OGT >15°C and MGT >20°C. However, above definition is ambiguous and questionable to emphasise a MGT as an indicator of how well an organism is adapted to the cold habitat, because the temperature limits have been arbitrarily selected and do not correspond to clear separation of biological processes or environmental conditions. The terms stenothermal and eurythermal have also been proposed for organisms that grow over narrow and wide temperature ranges, respectively. “True” or “obligate” psychrophiles can be classified as stenothermal psychrophiles, whereas “facultative” psychrophiles or “psychrotrophs” can be classified as eurythermal psychrophiles (Russell 2006). Such a definition takes into account the fact that cold-adapted microorganisms with wide range of growth temperature are much more abundant in cold environments, probably because they can tolerate a broad range of temperature. Although concept of stenothermal and eurythermal psychrophiles seems more concise, classical concepts are still widely accepted. In this review, we will also use the general term psychrophiles with narrower range of growth temperature.

## 2. Cold-adapted fungi and their living strategies

Current knowledge about the cold-adapted fungi has shown that even the most extreme cold habitat harbours enormously diverse and metabolically active fungal communities that constitute a large portion of low-temperature biodiversity. To survive in the harsh environments, fungal strains have developed series of living strategies and function as saprobes, symbionts, plant and animal parasites and pathogens to perform various ecological roles.

### 2.1. Fungal investigations in cold environments

Study on fungi in cold environments can be traced to the late nineteenth century (Bommer and Rousseau 1905; Brown 1906). Cold-adapted fungi belong to different phyla which have been reported from a wide variety of substrates from a broad geographical locations and diverse habitats.

The North and South Poles represent the coldest, driest and most inhospitable habitats in our planet. Paleomycological and paleoecological investigations have indicated that fungi have presented in Antarctica since at least the Permian period because diverse fossil fungi have been found from the Triassic and Jurassic Periods (Stubblefield and Taylor 1983; Taylor and Osborne 1996; Harper et al. 2012). In general, the fungal communities of Antarctic soils are dominated by filamentous ascomycetes and basidiomycete yeasts (Connell et al. 2008; Arenz and Blanchette 2011). Filamentous basidiomycetes that are typically known as wood-decay fungi in temperate ecosystems have been very rarely isolated in Antarctica (Blanchette et al. 2004; Ludley and Robinson 2008). Fungi in Zygomycetes are also isolated with some frequency in Antarctic (Lawley et al. 2004). Although the fungi in Chytridiomycota were considered to be not frequently encountered, they were isolated from Antarctic lakes and ponds with improved method (Paterson 1973). Bridge and Spooner (2012) listed over 400 fungal genera and more than 1000 species that had been reported from Antarctic regions and suggested that “fungi may be the most diverse biota in the Antarctica”.

The total number of known fungal species in the Arctic is more than 4350. Timling et al. (2014) sampled soils in paired plots along the North American Arctic Transect, which spans all five bioclimatic subzones of the Arctic. A total of 7834 fungal clones were grouped into 1834 operational taxonomic units (OTUs) which were assigned into 8 phyla, 24 classes, 75 orders, 120 families and 214 genera. Ascomycota dominated the communities, followed by Basidiomycota. Among 75 families of Ascomycota, members from Verrucariaceae were most abundant. Among 36 families of Basidiomycota, Inocybaceae was the most abundant. Five families in Chytridiomycota and one family in each of Zygomycota, Glomeromycota, Blastocladiomycota and Neocallimastigomycota were detected, while Cryptomycota were only identified at the phylum level.

Fungi isolated from deep seas were first reported nearly 50 years ago (Roth et al. 1964), then a great number of fungi including some novel species have been published by both conventional culture-dependent (Raghukumar et al. 2004; Gadanho and Sampaio 2005; Damare et al. 2006; Nagahama et al. 2006; Le Calvez et al. 2009; Singh et al. 2010) and culture-independent methods (Lai et al. 2007; Le Calvez et al. 2009; Nagano et al. 2010). Le Calvez et al. (2009) reported striking differences in their comparison of deep-sea fungal diversity assessed by culture-dependent and culture-independent methods. Generally, ascomycetes can be frequently and easily isolated in deep-sea environments, but chytrids and other basal fungi may be missed because many chytrids can not be isolated on solid growth media, especially those to which chloramphenicol has been added (Gleason and Marano 2011).

The Qinghai–Tibet Plateau, often called the “world’s roof” or “the third pole”, is the highest and largest low-latitude region with permafrost and glaciers in the world. The high elevation and low latitude make the Qinghai–Tibet Plateau a unique alpine ecosystem which possesses substantial new fungal species. Early mycology-based investigations mainly focused to macrofungi. Mao and Jiang (1993) investigated the macrofungi with commercial values in Tibet and described that 588 species belonged to 46 families, 173 genera. Compared with the polar regions, cold-adapted fungi in the Qinghai–Tibet Plateau were relatively poorly studied with the exception of the Chinese caterpillar fungus. A systemic survey on cold-adapted fungi had been conducted by Wang et al. (2015), with more than 1400 fungal strains isolated and 150 species including 6 new species identified and described from glaciers on the Qinghai–Tibet Plateau. Among those species, *Phoma sclerotioides* and *Pseudogymnoascus pannorum* were the most dominant species. Hassan (2015) isolated 77 fungal strains representing 24 fungal genera from Batura Passu and Siachen Glaciers in Hindu Kush and Karakoram mountains in Pakistan.

Except for being common in alpine, Arctic, Antarctic and deep-sea environments, cold-adapted fungi are also found in man-made habitats such as refrigerated environments where temperatures were often at or below 0°C. Many fungi isolated from the cold environments are cosmopolitan, i.e. *Phoma herbarum* is frequently isolated from the permafrost (Singh et al. 2006), as well as from soils of the temperate and subtropical zones (Domsch et al. 1980). Most of the cold-adapted fungi have wide growth temperature ranges. *Epicoccum purpurascens* is an example to have a minimum growth temperature at −3 to −4°C, an optimum at 23–28°C and a maximum temperature for growth at 45°C (Domsch et al. 1980). Only a few cold-adapted fungi with narrow growth temperature ranges were reported (Table 1).

**Table 1.** Cold-adapted fungi with narrow growth temperatures (psychrophilic fungi).

Taxa	Growth temperature	Location/Substrate/Host	Reference
*Mucor strictus*	OGT below 10°C	Alpine soils	Schipper (1967)
*Coprinus psychromorbidus*	OGT between −8 and −3°C	Plant pathogen infecting cereals (winter wheat, oats), grass and conifers	Traquair and Smith (1982)
*Typhula ishikariensis*	OGT at 10°C		Hoshino et al. (1998)
*Typhula incarnata*	OGT between 5 and 10°C		Nakajima and Abe (1994)
*Microdochium nivale*	OGT between 15 and 18°C		
*Sclerotinia borealis*	OGT between 4 and 10°C		Hoshino et al. (2010)
*Rhodotorula himalayensis*	–	Soils of Himalayan mountain ranges	Shivaji et al. (2008)
*Phoma herbarum*	OGT at 4°C	Endophytic fungi of trees	Moghaddam and Soltani (2014)
*Humicola marvinii*	OGT below 15°C, MGT at 20°C	Soils in the Maritime Antarctic	Richard et al. (1997)
*Pseudogymnoascus destructans*	OGT at 4°C	Hibernant bats	Gargas et al. (2009)
*Pseudogymnoascus pannorum*	OGT at 5°C	Soil and litter	Flanagan and Scarborough (1974)
*Thelebolus microsporus*	–	Pangong Lake, Himalayan region	Anupama et al. (2011)
*Mrakia robertii*		Antarctic and alpine soils	
*Mrakia blollopis*	MGT below 20°C		Robin et al. (2010)
*Mrakiella niccombsiis*			
*Mrakiella cryoconiti*	MGT below 20°C	Northern Siberian glacier sediment	Margesin and Fell (2008)
*Mrakia psychrophila*	OGT 10, MGT18	Antarctic soils	Xin and Zhou (2007)
*Rhodotorula psychrophila*	OGT below 15°C	Alpine soils	Margesin et al. (2007)
*Tetracladim ellipsoideum*	OGT below 15°C	Glacier soils in Qinghai–Tibet Plateau	Wang et al. (2015)
*Tetracladim globosum*	OGT below 15°C		
*Tetracladium psychrophilum*	OGT below 15°C, MGT below 20°C		

OGT: Optimum growth temperature; MGT: maximum growth temperature.

### 2.2. Living strategies of cold-adapted fungi

#### 2.2.1. Saprobes

Fungi are pivotal for the cycling of carbon and nutrients in cold ecosystems and the saprotrophic fungi actively decompose organic matters through excretion of a variety of hydrolases. A group of cold-adapted fungi decompose woody structures and artefacts left by the early polar explorers in Antarctic. Blanchette et al. (2004) first reported an unusual form of soft rot decay caused by *Cadophora* species which can cause degradation of the historic huts and artefacts. This type of decay has subsequently been found to be prevalent in historic woods and in soils from the immediate vicinity of the huts at many Antarctic locations and variety of filamentous fungi and yeasts such as *Cadophora, Cladosporium, Cryptococcus* and *Geomyces* species were discovered with a high frequency (Arenz et al. 2006; Arenz and Blanchette 2009; Blanchette et al. 2010). Although there are few woody plants on the Antarctic continent, researches provide strong evidence that Antarctic fungi are able to colonise and degrade-introduced wood and other organic materials (Blanchette et al. 2004, 2010). Held et al. (2005) found that Antarctic summer above 0°C and 75% relative humidity occurred for many weeks, which are conducive for fungal growing inside the historic huts on Ross Island. Air sampling of fungal spores in the interior and exterior areas of the historic huts demonstrated that *Antarctomyces psychrotrophicus Cladosporium cladosporioides, Geomyces* sp. and *Pseudeurotium desertorum* dominated the air environments within the huts and fungal spores are widespread particularly in the interiors of the huts (Duncan et al. 2010). Blanchette et al. (2010) reported that large numbers of degradative fungi colonised the exterior wood of Shackleton’s Cape Royds hut, presumably due to large carbon and nutrient input from the historic materials as well as introductions from the penguin colony nearby. Both culture-dependent and culture-independent methods demonstrated that low temperature may not adversely affect these fungal species unless they were out-competed by new arrivals or unfavourable changes in ecosystem domination occurred (Farrell et al. 2011).

A special group of fungi extensively studied in Antarctic is the rock-inhibiting fungi, which constitute an important part of epilithic and endolithic communities (Selbmann et al. 2014). To cope with the harsh conditions of the outside, these fungi adopt a particular lifestyle and live as so-called cryptoendolithic and chasmoendolithic (Golubic et al. 1981) in microscopic niches inside the rocks (Nienow and Friedmann 1993; De Los Ríos et al. 2002). These fungi living on or within the rocks share similar morphological and physiological characteristics, such as slow expanding cauliform-like colony, barely differentiated structures and the presence of thick and heavily pigmented cell walls. Their morphological features are suitable to withstand harsh environmental conditions; for example, the meristematic growth allows them to keep optimal volume/surface ratio in order to minimise exchange with external stressors. Another remarkable character is the production of extracellular polymeric substances (EPS) which is involved in the protection against cycles of desiccation, freezing and thawing (Selbmann et al. 2002, 2005); EPS surround fungal structures (hyphae and conidia), as observed both in nature and in culture, create and maintain the proper micro-environmental conditions in the biofilms inside the rocks buffering pH and nutrient availability fluctuations (De Los Ríos et al. 2002). The Antarctic genus *Cryomyces* with highly pigmented structures showed a higher resistance to UV radiation compared with an Antarctic strain of filamentous species *Arthrobotrys ferox*. However, a European strain of *Arthrobotrys oligospora* is more sensitive to UV than *A. ferox* (Zucconi et al. 2002; Onofri et al. Forthcoming). Most of the Cryptoendolithic black fungi show an extreme simplification of their structures and some are even unable to produce complex and well-differentiated reproductive structures, and propagules are produced directly from disarticulation of toruloid pre-existing hyphae. Some of them are mostly yeast-like organised and can formally conclude their life cycles with the production of a single cell being itself a resistant propagule. These high levels of simplification match very well with the prohibitive environment they colonised where the climatic conditions for active life happen just few days a year.

Nearly 90% of the volume of marine habitats is below 5°C and the deep seas which constitutes the largest unexplored habitat on our planet often have temperatures from −1 to −4°C (Frisvad 2008). Deep-sea sediments appear to be one of the potential niches for hidden fungal diversity and the fungal roles in the deep-sea sediments have remained neglected mainly due to the fact that they are not easily observed (Damare et al. 2008). Increased sighting of fungi after treating the deep-sea sediments with chelating agent suggested that fungi in deep-sea sediments may be involved in humic aggregate formation (Liu et al. 2002). Hyphal sheaths often have an adhesive role in the attachment of fungal mycelium to surface and particle entrapment (Hyde et al. 1986). Through concealing inside the microaggregates, which prevent the extracellular enzymes from diffusing away from the cells secreting it, these humic–enzyme complexes have an important role to play in overall nutrient dynamics of the sediments (Burns 1978). Protease is one of the several extracellular enzymes playing a major role in their nutritional requirements. Damare et al. (2006) demonstrated that 11% of the total fungi isolated from deep-sea sediments of Central Indian Basin showed low temperature-active protease production. The proteases from deep-sea fungi could be a potential source in the search for detergent enzymes (Raghukumar et al. 2009). Fungal polygalacturonases (PGases) are useful enzymes for clarification of fruit juices in the food industry. Two novel and active endopolygalacturonases at 0–10°C were purified from the culture supernatant of a deep-sea yeast *Cryptococcus* sp. which was isolated from the Japan Trench at a depth of 4500–6500 m (Miura et al. 2001; Abe et al. 2006). The hydrolytic activity of PGases remained almost unchanged up to a hydrostatic pressure of 100 MPa at 24°C. Interestingly, this strain was tolerant to CuSO_4_ up to a concentration of 50 mM and showed high activity of superoxide dismutase, an enzyme responsible for scavenging superoxide radicals (Abe et al. 2001).

#### 2.2.2. Plant mutualists

Lichen and mycorrhiza are ubiquitous fungal mutualism form and also dominant in the cold habitats. Mycorrhizal fungi play an important role in the functioning of cold ecosystems, where low water and nutrient availability restrict plant growth and productivity (Timling and Taylor 2012). The complex mycorrhizal structure, consisting of plant root and fungal hyphae, could enlarge the surface area for absorbing water and nutrients from soil, explore for nutrients more extensively than vascular-plant roots and mobilise organically bound nutrients. The fungal association provides the fungus direct access to the plant’s carbohydrates, while the plants gain benefits from improved mineral and water absorption through the fungal mycelia. It is estimated that 86% of the N obtained by Arctic plants is via mycorrhizal, notably ectomycorrhizal (ECM) fungi (Hobbie and Hobbie 2006). EMF genera were the most frequent and species-rich associated with plants in the Arctic (Bjorbaekmo et al. 2010; Deslippe et al. 2011; Geml et al. 2012). Coupled with previous sporocarp collections (Gardes and Dahlberg 1996), the frequently occurred fungal genera that either lack or produce sporocarps are *Thelephora, Sebacina* and *Clavulina*. EMF resulted from analysis of root tips and soil clones in Arctic revealed their extremely high richness (Bjorbaekmo et al. 2010; Geml et al. 2012), comparing with previous estimation based on surveys of above-ground ECM sporocarps. A large number of OTUs were detected in Arctic, with 137 OUTs on the roots of *Dryas octopetala* along a latitudinal gradient from Southern Norway to Svalbard (Bjorbaekmo et al. 2010), 73 ECM basidiomycete OTUs in soils on Svalbard (Geml et al. 2012) and 224 OTUs in the roots of three co-occurring species in the Low Arctic (Walker et al. 2011).

Lichens, the intimate and stable association between fungi and green algae/cyanobacteria, are the visually prominent life forms in most extreme ecosystems. In harsh environments, they have the general ability to photosynthesise at sub-zero temperatures, tolerance to low water potentials and high radiation levels, and the ability to utilise sublimed water from ice or air (Green et al. 1999). Lichens in the Antarctic ecosystems have been studied thoroughly. Some of the Antarctic species have a very wide geographic distribution and can be found in habitats of the extreme North or in high mountains (Sancho et al. 1999; Øvstedal and Lewis Smith 2001). Communities of chasmoendolithic lichens are common in granitic rocks. Cells of both algal and fungal symbionts occupy fissures and cracks of the lithic substrate with no clear heteromerous structure (De Los Ríos et al. 2005a). This kind of growth has been recognised and characterised for two species of *Lecidea* in Granite Harbour area (De Los Ríos et al. 2005b). The widespread distribution of this life-form in ice-free areas in Antarctica could be favoured by the high frequency of rock-freeze-fracturing phenomena (Cowan and Tow 2004). Remarkably, lichen associations are able to integrate photoprotective systems which provide higher resistance to oxidative damages than alga or fungus alone (Kranner et al. 2005). An increase of phenolic compounds content under UV exposition, which may prevent UV penetration into lichen thallus, as well as play a protective role as antioxidants, has also been observed (Buffoni Hall et al. 2002).

#### 2.2.3. Endophytes

Endophytic fungi live inside healthy plants and synthesise an array of secondary metabolites that interact with and benefit their hosts. Studies dealt with fungal endophytes in cold ecosystems revealed high species diversity. Moghaddam and Soltani (2014) provided information on an endophytic association between bioactive cold-adapted fungi and trees in *Cupressaceae* living in temperate to cold habitats. The endophytic fungi from healthy foliar tissues of *Cupressus arizonica, Cupressus sempervirens* and *Thuja orientalis* (*Cupressaceae, Coniferales*) were identified, including 23 endophytic fungi in *Dothideomycetes*. All of these fungi isolated at 4°C were able to synthesise secondary metabolites at this temperature. The evergreen *Cupressaceae* plants may benefit from their psychrophilic endophytic fungi during cold stress. Cold-adapted endophytic fungi associated with five dominant plant species were collected from the Baima Snow Mountain (altitude 4000–4300 m), Southwest China (Li et al. 2012). Forty-three taxa were identified based on the morphological characteristics and Internal transcribed spacer (ITS) sequences, of which *Cephalosporium, Sirococcus, Penicillium* and *Aspergillus* were the dominant genera. Growth temperature tests indicated that 75% of the isolates from the Baima Snow Mountain were psychrotrophs and 14% were the transitional type between psychrotrophs and mesophiles, suggested that the endophytes from the Baima Snow Mountain possess a remarkable ability to adapt to cold environments.

#### 2.2.4. Parasites

Fungal parasites are normally specialised microfungi attacking plants, other fungi and animals including human. Fungal diseases on plants are common in cold environments, such as rust fungi *Melampsora* that commonly cause mortality of willows (Parmelee 1989; Smith et al. 2004) and smuts (Ustilaginales) that parasitise plants of Cyperaceae (Scholler et al. 2003) in Arctic.

In higher latitude and high altitude regions, where winter is longer and harsh, a deep, persistent layer of snow insulates the roots and crowns of winter cereals, grasses, forages, ornamentals and shrubs, protecting them from winter injuries. However, the dark, humid environmental conditions are of favourite for the development of cold-adapted microorganisms. Snow moulds cause serious diseases of snow-covered plants when the temperature at the snow-plant interface remains around 0°C. Snow mould are taxonomically diverse and can attack not only perennial grasses and winter cereals, turf grasses and flowers, but also woody plants, such as first-year pine seedlings or the lower parts of tree branches under snow and occur commonly in high latitude regions. Generally, phytopathogenic fungi can survive in the form of spores and sclerotia, to concur the winter harsh condition, and develop mycelia under the snow cover and resume infection the following season (Smith 1993). Four different snow mould diseases caused by soil-borne fungi are pink snow mould (*Microdochium* [*Fusarium*] *nivale*), speckled snow mould (*Typhula idahoensis, Typhula ishikariensis* and *Typhula incarnata*), snow scald (*Myriosclerotinia borealis*) and snow rot (*Pythium iwayami* and *Pythium okanoganense*). Pink snow mould is the most widespread, occurring on wild grasses, lawns and winter wheat, but less destructive than speckled snow mould. In addition to speckled snow mould, *T. incarnata* is often found causing a root and crown rot of wheat and barley in the absence of snow cover (Matsumoto 2009).

## 3. Representative cold-adapted fungal species

### 3.1. Pseudogymnoascus destructans, *a fatal pathogen causes bat white-nose syndrome*

*P*. *destructans* (formerly known as *Geomyces destructans*) is a psychrophilic fungus that causes white-nose syndrome (WNS) of hibernating and cave-roosting bats, a fatal disease that has devastated bat populations in parts of the United States and Canada. A visually conspicuous white fungus grows on the face, ears or wings of stricken bats; infiltration of the hyphae into membranes and tissues leads to severe damage (Meteyer et al. 2009). Bats that exhibit WNS have little or no fat reserves, which are essential for their survival throughout and after hibernation (Blehert et al. 2009). Although recent work on fungi associated with bat hibernacula uncovered many species of *Geomyces* and allies, including *Geomyces, Gymnostellatospora* and *Pseudogymnoascus* in the family *Pseudeurotiaceae, P. destructans* was demonstrated as unique pathogen of bats. The closest relatives of *P destructans* are members of the *Pseudogymnoascus roseus* species complex. There are no species phylogenetically close related to *P. destructans* providing evidence to support the hypothesis that this pathogen is non-native and invasive in eastern North America. Additional sampling from other regions of the world is necessary to better understand the evolution and biogeography of this important and diverse group of fungi (Minnis and Lindner 2013).

### 3.2. Ophiocordyceps sinensis, *a valued fungus as the traditional Chinese medicine*

The Chinese caterpillar fungus, *O*. *sinensis*, the so-called winter worm, summer grass in Chinese literature, is one of the most valued Chinese traditional medicinal fungi (Zhang et al. 2012). This fungus exhibits extremely high host specificity and colonises ghost moth caterpillars (*Thitarodes* spp.), making a parasitic complex that comprises the remains of the caterpillar and fungal sexual stroma. They are distributed in alpine regions of the Tibetan Plateau between 3000 and 5000 m as around the snow line and *O. sinensis* has an OGT at 15–18°C, but it can also survive at the temperature lower than −40°C in winter. Diverse bioactive ingredients and extensive medicinal effects (Chen et al. 2010; Lo et al. 2013) of Chinese cordyceps have been found and they have been applied to treat a variety of ailments including cancer, impotence and fatigue (Stone 2008). Owing to the medicinal, economic, social and ecological importance, and the limited distribution mainly in China, *O. sinensis* had been nominated as the national fungus of China (Zhang et al. 2012).

The spatial patterns of genetic variation of Chinese cordyceps including both the parasitoidal fungus *O. sinensis* and its host insects indicated that *O. sinensis* and its host insects originated at similar divergence time and geographic regions in southern Tibet/Yunnan, followed by range expansion to their current distributions. Cophylogenetic analyses revealed a complex evolutionary relationship between *O. sinensis* and its host insects and both historical and contemporary events have played important roles in the phylogeography and evolution of the *O. sinensis*-ghost moth parasitoidism on the Tibetan Plateau (Zhang et al. 2014). *O. sinensis* is adapted to cold temperature with putative antifreeze proteins and mechanisms for increasing lipid accumulation and fatty acid unsaturation (Xiao et al. 2013). Recently, 31 strains of *O. sinensis* representing nearly all of its geographic range were analysed, and the results exhibited latitude-based population divergence and nature selection for population inhabitation towards higher altitudes on the Qinghai–Tibetan Plateau (Xia et al. 2017).

### 3.3. Mrakia psychrophila, *a fungus revealing cold adaptation mechanism*

*Mrakia psychrophila* was first described by Xin and Zhou (2007) from soil samples collected from Fildes Peninsula, Antarctica and its optimum and MGTs are at 10 and 18°C, respectively. The first integrated study on the genomic, transcriptomic and proteomic perspectives of *M. psychrophila* provided insights into the cold adaptation mechanism of psychrophilic fungi (Su et al. 2016). The comparative genomic analysis with that of other psychrophilic, thermophilic and mesophilic fungi indicated that *M. psychrophila* had a specific codon usage preference, especially for codons of Gly and Arg and its major facilitator superfamily transporter gene family was expanded. Transcriptomic analysis revealed that genes involved in ribosome, biosynthesis of unsaturated fatty acid and glycerol and energy metabolism were upregulated at 4°C, indicated that cold adaptation of *M. psychrophila* was mediated by synthesising unsaturated fatty acids to maintain membrane fluidity and accumulating glycerol as a cryoprotectant. In the meanwhile, genes involved in unfolded protein binding, protein processing in the endoplasmic reticulum, proteasome, spliceosome and mRNA surveillance were upregulated at 20°C, indicated that the death of *M. psychrophila* above 20°C might be caused by an unfolded protein response.

### 3.4. P. pannorum, *a source for novel metabolites*

*P*. *pannorum* is a soil-inhibiting fungus often associated with cold temperatures. It has been isolated from Arctic permafrost as well as the soils of Antarctica, glacier bank soils in some Asian countries (Deshmukh 2002; Arenz et al. 2006; Ozerskaya et al. 2008). This fungus maintains cell and membrane function at low temperatures by elevating levels of unsaturated fats and compounds with cryoprotectant properties such as trehalose and various polyols at low temperature (Finotti et al. 1996; Hayes and Mark 2012). *P. pannorum* is a slow-growing cold-adapted fungus, exhibiting growth below 0°C to as low as −20°C. Strains recovered from Antarctic cryopegs germinate at −2°C 2–3 weeks after inoculation. The fatty acid composition and metabolism of this species changed in response to environmental temperatures. Cultures isolated from different places exhibited morphological variations and different rates of glucose and lipid utilisation (Finotti et al. 1993, 1996).

*P. pannorum* produces a range of extracellular hydrolases including lipase, chitinase, urease and amylase (Hayes and Mark 2012). Some strains of *P. pannorum* isolated from cold habitats produce abundant cold-adapted α-amylases, which indicates its significant role as a desirable candidate in food processing. He et al. (2017) cloned the correlated gene AmyA1, and heterologously expressed it in *Aspergillus oryzae*. AmyA1 was also immobilised on magnetic nanoparticles and characterised. The detailed report of the enzymatic properties of AmyA1 gave new insights into fungal cold-adapted amylase and application study showed potential value of AmyA1 in the food and starch industries. Some bioactive metabolites also have pharmaceutical potentials, such as pannomycin, structurally similar to a compound known to inhibit the ATPase, SecA, in the bacterial translocase pathway (Parish *et al*. 2009). Additional metabolites have been isolated from *P. pannorum* including antimicrobial asterric acid derivatives called “geomycins”, which is active against *Aspergillus fumigatus*, as well as Gram-positive and Gram-negative bacteria (Li et al. 2008).

## 4. Conclusions

Low temperature environments harbour large numbers of fungal species which have great ecological and physiological significance. Cold-adapted fungi are important resources for discovery of novel secondary metabolites and enzymes. There are limited fungi that have been successfully cultured from cold environments. Improved culturing methods and growth media are needed in order to isolate and culture diverse fungi from these environments, including those with unique physiologies to adapt to extreme habitats. Similarly, it is necessary to improve culture-independent methods for the enhanced detection of cold-adapted fungi, especially the development of better molecular markers for investigating true fungal diversity. The origination and evolution of some representative species that cause ecological crisis or have economic importance should be investigated in depth. Ecological function and adaptation mechanism of certain species will provide comprehensive understanding of cold-adapted fungi.

## References

[CIT0001] Abe F, Minegishi H, Miura T, Nagahama T, Usami R, Horikoshi K. 2006 Characterization of cold-and high-pressure-active polygalacturonases from a deep-sea yeast, *Cryptococcus liquefaciens* strain N6. Biosci. Biotechnol.Biochem. 70:296–299. 16428855 10.1271/bbb.70.296

[CIT0002] Abe F, Miura T, Nagahama T, Inoue A, Usami R, Horikoshi K 2001 Isolation of a highlycopper-tolerant yeast, *Cryptococcus* sp., from the Japan Trench and the induction of superoxide dismutase activity by Cu_2+_ . Biotechnol Lett. 23:2027–2034.

[CIT0003] Anupama PD, Praveen KD, Singh RK, Kumar S, Srivastava AK, Arora DK. 2011 A psychrophilic and halotolerant strain of *thelebolus microspores* from Pangong Lake, Himalaya. Mycosphere. 2:601–609.

[CIT0004] Arenz BE, Blanchette RA 2009 Investigations of fungal diversity in wooden structures and soils at historic sites on the Antarctic Peninsula. Can J Microbiol. 55:46–56. 19190700 10.1139/W08-120

[CIT0005] Arenz BE, Blanchette RA 2011 Distribution and abundance of soil fungi in Antarctica at sites on the Peninsula, Ross Sea Region and McMurdo dry valleys. Soil Biol Biochem. 43:308–315.

[CIT0006] Arenz BE, Held BW, Jurgens JA, Farrell RL, Blanchette RA 2006 Fungal diversity in soils and historic wood from the Ross Sea Region of Antarctica. Soil Biol Biochem. 38:3057–3064.

[CIT0007] Bjorbaekmo MFM, Carlsen T, Brysting A, Vrålstad T, Høiland K, Ugland KI, Geml J, Schumacher T, Kauserud H. 2010 High diversity of root associated fungi in both alpine and arctic *Dryas octopetala* . BMC Plant Biology. 10:244. 21070665 10.1186/1471-2229-10-244 PMC3095326

[CIT0008] Blanchette RA, Held BW, Arenz BE, Jurgens JA, Baltes NJ, Duncan SM, Farrell RL 2010 An Antarctic hot spot for fungi at Shackleton’s historic hut on Cape Royds. Microb Ecol. 60:29–38. 20386896 10.1007/s00248-010-9664-z

[CIT0009] Blanchette RA, Held BW, Jurgens JA, McNew DL, Harrington TC, Duncan SM, Farrell RL 2004 Wood destroying soft-rot fungi in the historic expedition huts of Antarctica. Appl Environ Microbiol. 70:1328–1335. 15006750 10.1128/AEM.70.3.1328-1335.2004 PMC368374

[CIT0010] Blehert DS, Hicks AC, Behr M, Meteyer CU, Berlowski-Zier BM, Buckles EL 2009 Bat white-nose syndrome: an emerging fungal pathogen? Science. 323:227. 18974316 10.1126/science.1163874

[CIT0011] Bommer E, Rousseau M 1905 Champignons in resultats du voyage du SY Belgica, expedition Antarctique Beige 1897-1899. Rapports Scientifiques Botanique pp. 1–5

[CIT0012] Bridge PD, Spooner BM 2012 Non-lichenised Antarctic fungi: transient visitors or members of a cryptic ecosystem? Fungal Ecololgy. 5:381–394.

[CIT0013] Brown RNR 1906 Antarctic botany: its present state and future problems. Scottish Geogr Mag. 22:473–484.

[CIT0014] Buffoni Hall RS, Bornman JF, Björn LA 2002 UV-induced changes in pigment content and light penetration in the fruticose lichen *Cladonia arbuscula* ssp. mitis. J. Photochem Photobiol B Biol. 66:13–20. 10.1016/s1011-1344(01)00270-6 11849978

[CIT0015] Burns RG 1978 Enzymes in soil: some theoretical and practical considerations In: Burns RG, editor. Soil Enzymes. London: Academic Press; p. 295–339.

[CIT0016] Chen JY, Sangwoo L, Cao YQ, Peng YQ, Winkler D, Yang DR. 2010 Ethnomycological use of medicinal Chinese caterpillar fungus, *Ophiocordyceps sinensis* (Berk.) G. H. Sung *et al*. (Ascomycetes) in northern Yunnan province, SW China. Inter J Med Mush. 12:427–434.

[CIT0017] Connell LB, Redman R, Craig SD, Scorzetti G, Iszard M, Rodriguez R 2008 Diversity of soil yeasts isolated from South Victoria Land, Antarctica. Microb Ecol. 56:448–459. 18253776 10.1007/s00248-008-9363-1

[CIT0018] Cowan DA, Tow LA 2004 Endangered Antarctic environments. Annu Rev Microbiol. 58:649–690. 15487951 10.1146/annurev.micro.57.030502.090811

[CIT0019] Damare S, Nagarajan M, Raghukumar C 2008 Spore germination of fungi belonging to *Aspergillus* species under deep- sea conditions. Deep-Sea Res I. 55:670–678.

[CIT0020] Damare S, Raghukumar C, Raghukumar S 2006 Fungi in deep-sea sediments of the Central Indian Basin. Deep-Sea Research I. 53:14–27.

[CIT0021] de Los Ríos A, Sancho LG, Grube M, Wierzchos J, Ascaso C 2005b Endolithic growth of two *Lecidea* lichens in granite from continental Antarctica detected by molecular and microscopy techniques. New Phytol. 165:181–190. 15720632 10.1111/j.1469-8137.2004.01199.x

[CIT0022] de Los Ríos A, Wierzchos J, Sancho LG, Green A, Ascaso C 2005a Ecology of endolithic lichens colonizing granite in continental Antarctica. The Lichenol. 37:383–395.

[CIT0023] De Los Ríos A, Wierzchos J, Sancho LG, Grube M, Ascaso C 2002 Microbial endolithic biofilms: a means of surviving the harsh conditions of the Antarctic. European Space Agency Publications Division, 518:219–222.

[CIT0024] Deshmukh SK 2002 Incidence of dermatophytes and other keratinophilic fungi in the glacier bank soils of the Kashmir valley, India. Mycologist. 16(4):165–167.

[CIT0025] Deslippe JR, Hartmann M, Mohn WW, Simard SW 2011 Long-term experimental manipulation of climate alters the ectomycorrhizal community of Betula nana in Arctic tundra. Glob Chang Biol. 17:1625–1636.

[CIT0026] Domsch K-H, Gams W, Anderson T-H 1980 Compendium of soil fungi. London: Academic Press.

[CIT0027] Duncan SM, Farrell RL, Jordan N, Jurgens JA, Blanchette RA 2010 Monitoring and identification of airborne fungi at historic locations on Ross Island, Antarctica. Polar Sci. 4:275–283.

[CIT0028] Farrell RL, Arenz BE, Duncan SM, Held BW, Jurgens JA, Blanchette RA 2011 Introduced and indigenous fungi of the Ross Island historic huts and pristine areas of Antarctica. Polar Biol. 34:1669–1677.

[CIT0029] Finotti E, Moretto D, Marsella R, Mercantini R 1993 Temperature effects and fatty acid patterns in *Geomyces* species isolated from Antarctic soil. Polar Biol. 13(2):127–130.

[CIT0030] Finotti E, Paolino C, Lancia B, Mercantini R 1996 Metabolic differences between two antarctic strains of *Geomyces pannorum* . Curr Microbiol. 32(1):7–10.

[CIT0031] Flanagan PW, Scarborough AM 1974 Physiological groups of decomposer fungi on tundra plant remains In: Holding AJ, Heal OW, MacLean SF Jr, Flanagan PW, eds. Soil organisms and decomposition in tundra. Stockholm, Sweden: Tundra Biome Steering Committee; p. 159–181.

[CIT0032] Frisvad JC 2008 Fungi in Cold Ecosystems In: Margesin R, Schinner F, Marx JC, Gerday C, eds.. Psychrophiles: from Biodiversity to Biotechnology. Springer-Verlag, Berlin Heidelberg; p. 137–156.

[CIT0033] Gadanho M, Sampaio JP 2005 Occurrence and diversity of yeasts in the mid-atlantic ridge hydrothermal fields near the Azores Archipelago. Microbial Ecology. 50:408–417. 16328655 10.1007/s00248-005-0195-y

[CIT0034] Gardes M, Dahlberg A 1996 Mycorrhizal diversity in arctic and alpine tundra: an open question. New Phytologist. 133:147–157.

[CIT0035] Gargas A, Trest MT, Christensen M, et al 2009 *Geomyces destructans* sp. nov. associated with bat white-nose syndrome. Mycotaxon. 108:147–154.

[CIT0036] Geml J, Kauff F, Brochmann C, et al 2012 Frequent circumarctic and rare transequatorial dispersals in the lichenised agaric genus *Lichenomphalia* (*Hygrophoraceae, Basidiomycota*). Fungal Biology. 116:388–400. 22385621 10.1016/j.funbio.2011.12.009

[CIT0037] Gleason FH, Marano AV 2011 The effects of antifungal substances on some zoosporic fungi (Kingdom Fungi). Hydrobiologia. 659:81–92.

[CIT0038] Golubic S, Friedmann EI, Schneider J 1981 The lithobiontic ecological niche, with special reference to microorganisms. J Sediment Petrol. 51:475–478.

[CIT0039] Green TGA, Schroeter B, Sancho L 1999 Plant life in Antarctica In: Pugnaire FI, Valladares F, editors. Handbook of functional plant ecology. New York: Marcel Dekker; p. 495–543.

[CIT0040] Harper CJ, Bomfleur B, Decombeix AL, Taylor EL, Taylor TN, Krings M 2012 Tylosis formation and fungal interactions in an early Jurassic conifer from northern Victoria Land, Antarctica. Rev Palaeobot Palynol. 175:25–31.

[CIT0041] Hassan H 2015 Isolation and characterization of psychrophilic fungi from Batura Passu and Siachen Glaciers, Pakistan Dissertation Department of Microbiology, Quaid-i-Azam University, Islamabad.

[CIT0042] Hassan N, Rafiq M, Hayat M, Shah AA, Hasan F 2016 Psychrophilic and psychrotrophic fungi: a comprehensive review. Rev Environ Sci Biotechnol. 15(2):147–172.

[CIT0043] Hayes, Mark A 2012 The *Geomyces* Fungi: ecology and distribution. BioScience. 62(9):819–823.

[CIT0044] He et al.. 2017 Functional expression of a novel α-amylase from Antarctic psychrotolerant fungus for baking industry and its magnetic immobilization. BMC Biotechnol. 17:22. 28245836 10.1186/s12896-017-0343-8 PMC5331696

[CIT0045] Held BW, Jurgens JA, Arenz BE, Duncan SM, Farrell RL, Blanchette RA 2005 Environmental factors influencing microbial growth inside the historic huts of Ross Island, Antarctica. Int Biodeterior Biodegrad.55(1): 45–53.

[CIT0046] Hobbie JE, Hobbie EA 2006 15N In symbiotic fungi and plants estimates nitrogen and carbon flux rates in Arctic tundra. Ecology. 87:816–822. 16676524 10.1890/0012-9658(2006)87[816:nisfap]2.0.co;2

[CIT0047] Hoshino T, Terami F, Tkachenko OB, et al 2010 Mycelial growth of the snow mold fungus *Sclerotinia borealis*, improved at low water potentials: an adaptation to frozen environment. Mycoscience. 51:98–103.

[CIT0048] Hoshino T, Tronsmo AM, Matsumoto N, Araki T, Georges F, Goda T, Ohgiya S, Ishizaki K 1998 Freezing resistance among isolates of a psychrophilic fungus, *Typhula ishikariensis*, from Norway. Proc. NIPR Symp. Polar Biol. 11:112–118.

[CIT0049] Hyde KD, Jones EBG, Moss ST 1986 Mycelial adhesion to surfaces In: Moss ST, ed. The Biology of Marine Fungi. Cambridge: Cambridge Univ. Press; p. 331–340.

[CIT0050] Kranner I, Cram WJ, Zorn M, Wornik S, Yoshimura I, Stabentheiner E, Pfeifhofer HW 2005 Antioxidants and photoprotection in a lichen as compared with its isolated symbiotic partners. Proc. Natl. Acad. Sci. USA. 102:3141–3146. 15710882 10.1073/pnas.0407716102 PMC549463

[CIT0051] Lai X, Cao L, Tan H, Fang S, Huang Y, Zhou S 2007 Fungal communities from methane hydrate-bearing deep-sea marine sediments in South China Sea. Isme J. 1:756–762. 18059498 10.1038/ismej.2007.51

[CIT0052] Lawley B, Ripley S, Bridge P, Convey P 2004 Molecular analysis of geographic patterns of eukaryotic diversity in Antarctic soils. Appl Environ Microbiol. 70:5963–5972. 15466539 10.1128/AEM.70.10.5963-5972.2004 PMC522059

[CIT0053] Le Calvez T, Burgaud G, Mahe S, Barbier G, Vandenkoornhuyse P 2009 Fungal diversity in deep sea hydrothermal ecosystems. Applied and Environmental Microbiology. 75:6415–6421. 19633124 10.1128/AEM.00653-09 PMC2765129

[CIT0054] Li HY, Shen M, Zhou ZP, et al 2012 Diversity and cold adaptation of endophytic fungi from five dominant plant species collected from the Baima Snow Mountain, Southwest China[J]. Fungal Diversity. 54(1):79–86.

[CIT0055] Li Y, Sun B, Liu S, Jiang L, Liu X, Zhang H, Che Y 2008 Bioactive Asterric Acid Derivatives from the antarctic ascomycete fungus *Geomyces* sp. J Nat Prod. 71(9):1643–1646. 18720971 10.1021/np8003003

[CIT0056] Liu H, Herbert HP, Fang HP 2002 Extraction of extracellular polymeric substances (EPS) of sludges. J. Biotechnol. 95:249–256. 12007865 10.1016/s0168-1656(02)00025-1

[CIT0057] Lo HC, Hsieh C, Lin FY, Hsu TH 2013 A systematic review of the mysterious caterpillar fungus *Ophiocordyceps sinensis* in Dong-Chong Xia Cao (Dong Chong Xia Căo) and related bioactive ingredients. Journal of Traditional and Complementary Medicine. 3:16–32. 24716152 10.4103/2225-4110.106538 PMC3924981

[CIT0058] Ludley KE, Robinson CH 2008 Decomposer, Basidiomycota in Arctic and Antarctic ecosystems. Soil Biol Biochem. 40:11–29.

[CIT0059] Mao XL, Jiang CP 1993 Economic macrofungi of tibet. Beijing Science and Technology Press， Beijing.

[CIT0060] Margesin R, Fell JW 2008 *Mrakiella cryoconiti* gen. nov., sp. nov., a psychrophilic, anamorphic, basidiomycetous yeast from alpine and arctic habitats. Int J Syst Evol Microbiol. 58:2977–2982. 19060092 10.1099/ijs.0.2008/000836-0

[CIT0061] Margesin R, Fonteyne PA, Schinner F, Sampaio JP 2007 *Rhodotorula psychrophila* sp. nov., *Rhodotorula psychrophenolica* sp. nov. and *Rhodotorula glacialis* sp. nov., novel psychrophilic basidiomycetous yeast species isolated from alpine environments. Int J Syst Evol Microbiol. 57:2179–2184. 17766895 10.1099/ijs.0.65111-0

[CIT0062] Margesin R, Miteva V 2011 Diversity and ecology of psychrophilic microorganisms. Res Microbiol. 162:346–361. 21187146 10.1016/j.resmic.2010.12.004

[CIT0063] Matsumoto N 2009 Snow molds: a group of fungi that prevail under snow. Minireview. Microbes Environ. 24(1):14–20. 10.1264/jsme2.me09101 21566348

[CIT0064] Meteyer CU, Buckles EL, Blehert DS, Hicks AC, Green DE, Shearn-Bochsler V 2009 Histopathologic criteria to confirm white-nose syndrome in bats. J Vet Diagn Invest. 21:411–414. 19564488 10.1177/104063870902100401

[CIT0065] Minnis AM, Lindner DL 2013 Phylogenetic evaluation of *Geomyces* and allies reveals no close relatives of *Pseudogymnoascus destructans*, comb. Nov., in Bat Hibernacula of Eastern North America. Fungal Biolo. 117(9):638–649. 10.1016/j.funbio.2013.07.001 24012303

[CIT0066] Miura T, Abe F, Inoue A, Usami R, Horikoshi K 2001 Purification and characterization of novel extracellular endopolygalacturonases from a deep-sea yeast, *Cryptococcus* sp. N6, isolated from the Japan Trench. Biotechnol Lett. 23:1735–1739.

[CIT0067] Moghaddam MSH, Soltani J 2014 Psychrophilic endophytic fungi with biological activity inhabit *Cupressaceae* plant family[J]. Symbiosis. 63(2):79–86.

[CIT0068] Morita RY 1975 Psychrophilic bacteria. Bacteriol Rev. 39(2):144–167. 1095004 10.1128/br.39.2.144-167.1975 PMC413900

[CIT0069] Nagahama T, Hamamoto M, Horikoshi K 2006 *Rhodotorula pacifica* sp. nov., a novel yeast species from sediment collected on the deep-sea floor of the north-west Pacific Ocean. Int J Syst Evol Microbiol. 56:295–299. 16403901 10.1099/ijs.0.63584-0

[CIT0070] Nagano Y, Nagahama T, Hatada Y, Nunoura T, Takami H, Miyazaki J, Takai K, Horikoshi K 2010 Fungal diversity in deep-sea sediments e the presence of novel fungal groups. Fungal Ecol. 3:316–325.

[CIT0071] Nakajima T, Abe J 1994 Development of resistance to *Microdochium nivale* in winter wheat during autumn and decline and the resistance under snow. Can J Bot. 72:1211–1215.

[CIT0072] Nienow JA, Friedmann EI 1993 Terrestrial lithophytic (rock) communities In: Friedmann EI, editor. Antarctic microbiology. New York: Wiley-Liss; p. 343–412.

[CIT0073] Onofri S, Zucconi L, Selbmann L, De Hoog GS, Barreca D, Ruisi S, Grube M Forthcoming 2007 Fungi from Antarctic desert rocks as analogues for Martian life In: Cockell CS editor. Microorganisms and martian environment. European Space Agency Special Publication， Chapter 6.

[CIT0074] Øvstedal DO, Lewis Smith RI 2001 Lichens of Antarctica and South Georgia. A guide to their identification and ecology. Studies in Polar Research. Cambridge, UK: Cambridge University Press; p. 411.

[CIT0075] Ozerskaya S, Kochkina G, Ivanushkina, Gilichinsky DA 2008 Fungi in Permafros ([Online-Ausg.]. ed.). Berlin: Springer; p. 85–95.

[CIT0076] Parish CA., de la Cruz M, Smith SK., Zink D, Baxter J, Tucker-Samaras S, Collado J, Platas G, Bills Gerald, Díez MT, et al 2009 Antisense-guided isolation and structure elucidation of pannomycin, a substituted cis-decalin from geomyces pannorum. Journal of Natural Products. 72(1):59 –62. 19102658 10.1021/np800528a

[CIT0077] Parmelee JA 1989 The rusts (*Uredinales*) of arctic Canada. Can J Bot. 67:3315–3365.

[CIT0078] Paterson RA 1973 The occurrence and distribution of some aquatic phycomycetes on Ross Island and the dry valleys of Victoria Land, Antarctica. Mycologia. 65:373–387.

[CIT0079] Raghukumar C, Damare SR, Muraleedharan UD 2009 A process for the production of low temperature-active alkaline protease from a deep-sea fungus.EP1692296.

[CIT0080] Raghukumar C, Raghukumar S, Sheelu G, Gupta S, Nagendernath B, Rao B 2004 Buried in time: culturable fungi in a deep-sea sediment core from the Chagos Trench, Indian Ocean. Deep Sea Research Part I. 51:1759–1768.

[CIT0081] Richard WN, Palm ME, Johnstone K, et al 1997 Ecological and physiological characterization of *Humicola marvinii*, a new psychrophilic fungus from Fellfield soils in the Maritime Antarctic. Mycologia. 89(5):705–711.

[CIT0082] Robin S, Hall T, Turchetti B, et al 2010 Cold-adapted yeasts from Antarctica and the Italian Alps – description of three novel species: *mrakia robertii* sp. nov., *Mrakia blollopis* sp. nov. and *Mrakiella niccombsii* sp. Nov. Extremophiles. 14:47–59. 19898737 10.1007/s00792-009-0286-7 PMC2797416

[CIT0083] Roth FJ, Orpurt PA, Ahearn DJ 1964 Occurrence and distribution of fungi in a subtropical marine environment. Can J Bot. 42:375–383.

[CIT0084] Russell NJ 2006 Antarctic microorganisms: coming in from the cold. Art Newspaper. 8(1):247–248.

[CIT0085] Sancho LG, Schulz F, Schroeter B, Kappen L 1999 Bryophyte and lichen flora of South Bay (Livingston Island: south Shetland Islands, Antarctica). Nova Hedwigia. 68:301–337.

[CIT0086] Schipper MA 1967 Mucor strictus hagem, a psychrophilic fungus, and *Mucor falcatus* sp.n. Antonie Van Leeuwenhoek. 33(2):189–195. 5298919 10.1007/BF02045550

[CIT0087] Scholler M, Schnittler M, Piepenbring M 2003 Species of *Anthracoidea* (*Ustilaginales, Basidiomycota*) on *Cyperaceae* in Arctic Europe. Nova Hedwig. 76:415–428.

[CIT0088] Selbmann L, De Hoog GS, Mazzaglia A, Friedmann EI, Onofri S 2005 Fungi at the edge of life: cryptoendolithic black fungi from Antarctic deserts. Stud. Mycol.. 51:1–32.

[CIT0089] Selbmann L, Onofri S, Fenice M, Federici F, Petruccioli M 2002 Production and structural characterization of the exopolysaccharide of the Antarctic fungus *Phoma herbarum* CCFEE 5080. Res Microbiol. 153:585–592. 12455706 10.1016/s0923-2508(02)01372-4

[CIT0090] Selbmann L, Zucconi L, Onofri S, Cecchini C, Isola D, Turchetti B, Buzzini P 2014 Taxonomic and phenotypic characterization of yeasts isolated from worldwide cold rock-associated habitats. Fungal Biology. 118:61–71. 24433677 10.1016/j.funbio.2013.11.002

[CIT0091] Shivaji S, Bhadra B, Rao RS, Pradhan S 2008 *Rhodotorula himalayensis* sp. nov., a novel psychrophilic yeast isolated from Roopkund Lake of the Himalayan mountain ranges, India. Extremophiles. 12:375–381. 18305897 10.1007/s00792-008-0144-z

[CIT0092] Singh P, Raghukumar C, Verma P, Shouche Y 2010 Phylogenetic diversity of culturable fungi from the deep-sea sediments of the Central Indian Basin and their growth characteristics. Fungal Diversity. 40:89–102.

[CIT0093] Singh SM, Puja G, Bhat DJ 2006 Psychrophilic fungi from Schirmacher Oasis, East Antarctica. Curr Sci. 90:1388–1392.

[CIT0094] Smith D 1993 Tolerance to freezing and thawing In: Jenninuns DH, editor. Stress Tolerance of Fungi. e w York: Marcel Dekker Inc; p. 147–171.

[CIT0095] Smith JA, Blanchette RA, Newcombe G 2004 Molecular and morphological characterization of the willow rust fungus, *Melampsora epitea*, from arctic and temperate hosts in North America. Mycologia. 96:1330–1338. 21148956

[CIT0096] Stone R 2008 Last stand for the body snatcher of the Himalayas? Science. 322:1182–1182. 19023056 10.1126/science.322.5905.1182

[CIT0097] Stubblefield SP, Taylor TN 1983 Studies of Paleozoic fungi. I. The structure and organization of Traquairia (Ascomycota). Amer J Bot. 70:387–399.

[CIT0098] Su Y, Jiang XZ, Wu WP, Wang MM, Hamid MI, Xiang MC, Liu XZ 2016 Provide insights into the cold adaptation mechanism of the obligate psychrophilic fungus *Mrakia psychrophila* . G3. 6(11):3603–3613. 10.1534/g3.116.033308 PMC5100859 27633791

[CIT0099] Taylor TN, Osborne JM 1996 The importance of fungi in shaping the paleoecosystem. Rev Paleobot Palynol. 90:249–262.

[CIT0100] Timling I, Taylor DL 2012 Peeking through a frosty window: molecular insights into the ecology of Arctic soil fungi. Fungal Ecol. 5:419–429.

[CIT0101] Timling I, Walker DA, Nusbaum C, Lennon NJ, Taylor DL 2014 Rich and cold: diversity, distribution and drivers of fungal communities in patterned-ground ecosystems of the North American Arctic. Molecular Ecology. 23:3258–3272. 24689939 10.1111/mec.12743

[CIT0102] Traquair JA, Smith DJ 1982 Sclerotial strains of *Coprinus psychromorbidus*, a snow mold basidiomycete. Canadian Journal of Plant Pathology. 4(1):27–36.

[CIT0103] Walker DA, Kuss P, Epstein HE, et al 2011 Vegetation of zonal patterned-ground ecosystems along the North America Arctic bioclimate gradient. Applied Vegetation Science. 14:440–463.

[CIT0104] Wang M, Jiang X, Wu W, et al 2015 Psychrophilic fungi from the world’s roof. Persoonia. 34:100–112. 26240448 10.3767/003158515X685878 PMC4510274

[CIT0105] Xia, et al 2017 *The caterpillar fungus, Ophiocordyceps sinensis*, genome provides insights into highland adaptation of fungal pathogenicity. Sci Rep. 7:1806. 28496210 10.1038/s41598-017-01869-z PMC5432009

[CIT0106] Xiao HU, Zhang YJ, Xiao GH, et al 2013 Genome survey uncovers the secrets of sex and lifestyle in caterpillar fungus[J]. Chinese Science Bulletin. 58(23):2846–2854.

[CIT0107] Xin MX, Zhou PJ 2007 J. Zhejiang Univ., Sci. B. 8(4):260–265. 17444601 10.1631/jzus.2007.B0260 PMC1838835

[CIT0108] Xin MX Zhou PJ 2007 Mrakia psychrophila sp. nov. a new species isolated from antarctic soil. j. zhejiang univ. sci. B. 8(4):260–265. 10.1631/jzus.2007.B0260 PMC1838835 17444601

[CIT0109] Zhang Y, Li E, Wang C, Li Y, Liu X 2012 *Ophiocordyceps sinensis*, the flagship fungus of China: terminology, life strategy and ecolog y. Mycology. 3:2–10.

[CIT0110] Zhang Y, Zhang S, Li Y, Ma S, Wang C, Xiang M, Liu X, An Z, Xu J, Liu X 2014 Phylogeography and evolution of a fungal-insect association on the tibetan plateau. Molecular Ecology. 23(21):5337–5355. 25263531 10.1111/mec.12940

[CIT0111] Zucconi L, Ripa C, Selbmann L, Onofri S 2002 Effects of UV on the spores of the fungal species *Arthrobotrys oligospora* and *A. ferox* . Polar Biol. 25:500–505.

